# Concordant X-linked hypophosphatemic rickets in monozygotic twins: diagnostic challenges and a novel genetic insight

**DOI:** 10.1530/EDM-25-0078

**Published:** 2025-09-19

**Authors:** Sara Ribeiro, Telma Moreno, Ana Varela, Gabriela Soares, Joana Queirós

**Affiliations:** ^1^ Department of Endocrinology, Diabetes and Metabolism, Unidade Local de Saúde de São João, Porto, Portugal; ^2^ Faculty of Medicine, Universidade do Porto, Porto, Portugal; ^3^ Genetics Department, Centro Hospitalar de Trás-os-Montes e Alto Douro, EPE, Vila Real, Portugal

**Keywords:** bone, rare diseases/syndromes, X-linked hypophosphatemic rickets (XLH)

## Abstract

**Summary:**

X-linked hypophosphatemic (XLH) is the most common inherited form of rickets, caused by inactivating mutations in the PHEX gene. Resultant overproduction of fibroblast growth factor 23 (FGF23) leads to renal phosphate wasting, reduced 1,25-dihydroxyvitamin D (1,25(OH)_2_D) levels, and impaired bone mineralization. We describe 26-year-old male monozygotic twins with lifelong skeletal deformities, short stature, and chronic bone pain. Despite hallmark features of rickets, both were misdiagnosed for decades and developed progressive functional impairment. Biochemical investigations revealed persistent hypophosphatemia, elevated alkaline phosphatase, reduced tubular maximum reabsorption of phosphate per glomerular filtration rate (TmP/GFR), normal calcium and parathyroid hormone, and inappropriately normal 1,25(OH)_2_D. Radiographs showed pseudofractures, consistent with osteomalacia. The twins were born from a triplet pregnancy; their dizygotic female sibling remained asymptomatic and biochemically normal. Genetic analysis revealed a novel *de novo* hemizygous deletion in exon 22 of PHEX, confirming the diagnosis of XLH. Both patients initiated conventional therapy with oral phosphate and calcitriol, resulting in notable clinical improvement, including restored ambulation and reduced pain. To our knowledge, this is the first documented case of phenotypically concordant XLH in monozygotic twins caused by a previously unreported PHEX mutation. The presentation underscores the risk of diagnostic delays in XLH, particularly in sporadic cases without family history, and highlights the value of early molecular testing in complex skeletal disorders. Timely recognition and treatment of XLH are essential to prevent irreversible complications and improve long-term outcomes, even when initiated in adulthood.

**Learning points:**

- XLH should be considered in patients with skeletal deformities, short stature, and recurrent dental abscesses.
- Diagnosis is frequently delayed due to variable phenotype and misdiagnosis as nutritional rickets or isolated orthopedic conditions.
- Biochemical findings of isolated hypophosphatemia with inappropriately normal 1,25(OH)_2_D levels should prompt evaluation for FGF23-mediated phosphate-wasting conditions.
- In cases without family history, genetic testing remains essential to confirm XLH and may reveal *de novo* mutations with clinical and research relevance.
- Conventional therapy with phosphate and calcitriol may lead to meaningful clinical improvement, including restored mobility, even in adults with long-standing disease.

This case contributes to the understanding of genotype–phenotype relationships in XLH, highlighting the potential value of twin studies in elucidating the genetic and non-genetic modifiers of disease expression.

## Background

X-linked hypophosphatemic (XLH) rickets is a rare genetic disorder caused by inactivating mutations in the *PHEX* (phosphate-regulating endopeptidase homolog, X-linked) gene, inherited in an X-linked dominant manner, with an estimated incidence of 3.9 per 100,000 live births (1).

Although the pathogenesis of XLH is complex and not fully elucidated, it primarily involves elevated circulating levels of fibroblast growth factor 23 (FGF23), secondary to PHEX inactivation. FGF23, produced mainly by osteocytes, induces renal phosphate wasting by downregulating the sodium–phosphate co-transporters NPT2a and NPT2c in the proximal renal tubules. In addition, FGF23 inhibits renal 1α–hydroxylase activity, reducing the conversion of 25-hydroxyvitamin D (25(OH)D) into its active form, 1,25-dihydroxyvitamin D (1,25(OH)_2_D), while simultaneously stimulating 24-hydroxylase, which enhances catabolism of active vitamin D. The resulting decline in 1,25(OH)_2_D levels impairs intestinal absorption of calcium and phosphate, further exacerbating hypophosphatemia and contributing to impaired skeletal mineralization (2). This defective mineralization manifests as rickets – characterized by inadequate mineralization of the growth plates in children – and osteomalacia, the impaired mineralization of mature bones, which may persist into or emerge in adulthood (1).

Although the phenotype can vary widely, clinical manifestations typically begin in early childhood. Common early signs include abnormal gait, lower limb deformities, poor growth velocity, craniosynostosis, and bone pain. Dental abscesses and periodontitis are also frequent. When unrecognized and untreated, progression into adulthood may be associated with more severe complications, such as enthesopathies, pseudofractures, osteoarthritis, spinal stenosis, and extraskeletal manifestations, including hearing loss and Arnold–Chiari malformations – resulting in significant functional limitations and impaired quality of life (2). Despite its genetic nature and early onset, XLH is frequently underdiagnosed or diagnosed late. Several factors contribute to this delay, including its broad phenotypic variability, the nonspecific nature of early symptoms, and frequent misdiagnosis as nutritional rickets or isolated orthopedic conditions. Furthermore, due to its multisystemic manifestations – affecting the skeletal, renal, dental, auditory, and neurological systems – patients are often assessed by different specialists. In the absence of coordinated, multidisciplinary care and effective communication among providers, the underlying metabolic cause may go unrecognized for years. Limited access to specialized laboratory and genetic testing further compounds the diagnostic challenge. Given its wide clinical spectrum and potential for long-term morbidity, early diagnosis and treatment of XLH are critical. Greater awareness across all healthcare disciplines, along with a collaborative, multidisciplinary approach, is essential to reduce diagnostic delays, initiate timely therapy, and improve long-term outcomes. We present a rare case of phenotypically concordant monozygotic twins with XLH caused by a novel pathogenic deletion in exon 22 of the *PHEX* gene, illustrating both the diagnostic complexity of sporadic presentations and the long-term consequences of delayed recognition and treatment.

## Case presentation

We report the cases of 26-year-old monozygotic male twins, born from a triplet pregnancy alongside a non-monozygotic female sibling who remains clinically unaffected. They were referred to Endocrinology by a hematologist investigating suspected polycythemia, which was ultimately ruled out. During this evaluation, both twins were found to have marked hypophosphatemia, raising suspicion for an underlying metabolic bone disorder.

**Figure 1:**
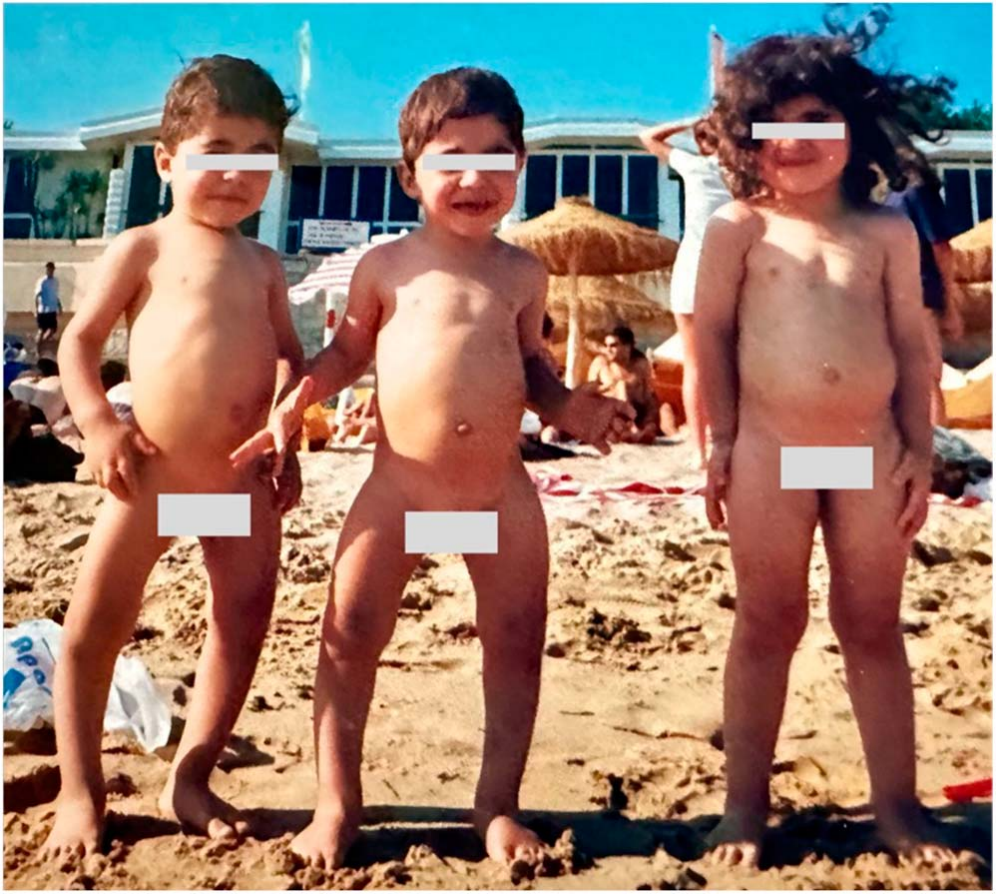
Photograph of the triplet siblings at age 4. The two monozygotic males (left and center) show visible bilateral lower limb bowing (genu varum), in contrast to their unaffected dizygotic sister (right).

Since early childhood (around age 2–3), both patients have exhibited lower limb deformities, short stature, and abnormal gait (Fig. 1). They were initially diagnosed with nutritional rickets and prescribed cholecalciferol, although follow-up was irregular and incomplete. Due to progressive limb bowing with bilateral genu varum, both underwent multiple orthopedic interventions starting at age 7, including femoral and tibial osteotomies with internal fixation, followed by surgical revisions up to age 12.

Twin 1 sustained a spontaneous femoral fracture at age 9, in the absence of significant trauma, leading to a provisional diagnosis of osteopetrosis. This hypothesis was never confirmed nor further investigated. Later, both twins were diagnosed with scoliosis and craniofacial dysmorphism. During adolescence, Twin 2 began experiencing recurrent headaches and was diagnosed with Arnold–Chiari type I malformation (age 17). He remains under neurosurgical surveillance without current indication for intervention. His brother, evaluated later, did not present with this anomaly.

By age 18, both developed bilateral coxarthrosis, chronic bone pain, and progressive loss of mobility. Both reported poor dentition since childhood, with multiple caries and recurrent dental abscesses. Despite evaluation by several specialties – including pediatrics, orthopedics, neurology, and physiatry – a unifying diagnosis had not been established.

There was no history of consanguinity or known family history of short stature, skeletal deformities, or fractures. The female triplet, their mother, and a maternal aunt had a history of early onset hypoacusis. An older sister and other family members, including the maternal uncle, were reported to be healthy and free of similar clinical features. The father had died at age 49 from alcohol-related complications.

At the time of referral to Endocrinology, at age 26, both twins presented with disabling lower extremity pain and severely impaired mobility. They had been taking daily cholecalciferol for the preceding 3 months, as advised by the hematologist, and used paracetamol or nonsteroidal anti-inflammatory drugs intermittently for bone and muscle pain. On physical examination, both had a height of 1.45 m (predicted adult height: 1.73 m). Twin 1 weighed 64 kg (BMI = 30.4) and Twin 2 weighed 70 kg (BMI = 33.0). Blood pressure measurements were 135/79 mmHg and 133/81 mmHg, respectively. Twin 1 was wheelchair-dependent, while Twin 2 ambulated with the aid of a walker. Both exhibited marked bilateral genu varum.

**Figure 2:**
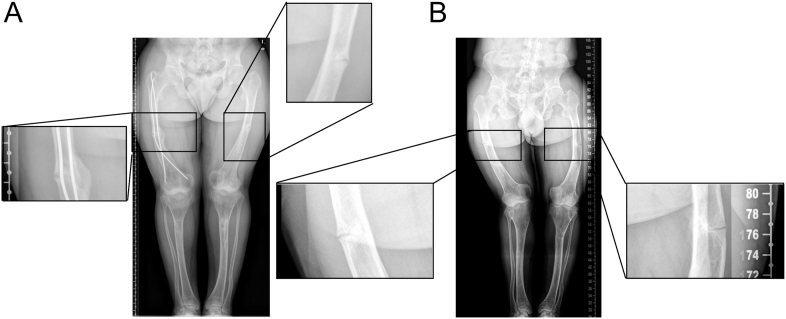
Radiographs of 26-year-old twin 1 (A) and twin 2 (B). The insets highlight diaphyseal lucencies with periosteal reaction, consistent with insufficiency fractures (pseudofractures) due to long-standing untreated hypophosphatemic rickets.

Biochemical analysis showed persistent hypophosphatemia, inappropriately normal 1,25(OH)_2_D level, and normal levels of parathyroid hormone, calcium, and 25-hydroxyvitamin D. Renal function was normal. The tubular maximum reabsorption of phosphate per glomerular filtration rate (TmP/GFR) was reduced and serum alkaline phosphatase was elevated (Table 1). Radiographs revealed multiple pseudofractures and bilateral coxarthrosis (Fig. 2). Renal ultrasound ruled out nephrocalcinosis and urolithiasis in both patients.

**Table 1:** Biochemical parameters of both twins at baseline and after 1 year of conventional treatment with oral phosphate and calcitriol. Marked hypophosphatemia and elevated alkaline phosphatase levels were observed before therapy. After 1 year, both patients demonstrated a modest improvement in serum phosphorus and biochemical parameters, consistent with a partial therapeutic response.

	Twin 1		Twin 2		Normal range
	Before treatment	1 year after treatment	Before treatment	1 year after treatment	
Hemoglobin, g/dL	17.8	17.1	18.0	17.2	13–19
Albumin, g/L	46.8	49.6	45.2	49.6	38–51
Creatinine, mg/dL	0.56	0.59	0.62	0.63	0.67–1.17
Serum phosphorus, mg/dL	1.1	1.4	1.2	1.4	2.7–4.5
Calcium, mg/dL	8.9	9.4	9.5	9.8	8.4–10.2
Magnesium, mg/dL	1.64	1.55	1.67	1.57	1.55–2.05
Alkaline phosphatase, U/L	188	135	162	125	30–120
PTH, pg/mL	43.1	67.7	43.7	71.2	10–68.3
25(OH)D, ng/mL	26	36	32	37	>30
1,25(OH)_2_D, pg/mL	64.7	-	60.2	-	15.7–90.1
Urinary creatinine, mg/dL	101.1	97.3	105.2	111.0	24–392
Urinary phosphorus, mg/dL	49.8	33.4	53.7	48.8	
TRP	74.9%	85.5%	73.6%	83.2%	85–95% (25)
TmP/GFR, mg/dL	0.82	1.20	0.88	1.17	2.6–3.8 (26)
Urinary calcium, mEq/24 h	2.4	2.8	2.2	2.6	5.0–15

PTH, parathyroid hormone; 25(OH)D, 25-hydroxyvitamin D; 1,25(OH)_2_D, 1,25-dihydroxyvitamin D; TRP, tubular reabsorption of phosphate; TmP/GFR, tubular maximum reabsorption of phosphate per glomerular filtration rate.

Molecular analysis identified a novel hemizygous deletion encompassing exon 22 of the *PHEX* gene: [c.(2,147+1_2,148−1)_(*408_?)del p.?]. Although this specific variant has not been previously reported in the literature, overlapping deletions in exon 22 have been described in *PHEX*-specific variant databases and classified as pathogenic. The mutation was present in both twins (confirmed to be monozygotic) and absent in their mother, confirming a *de novo* origin. Due to their history of hypoacusis, the mother and female triplet underwent further genetic evaluation and were found to carry a pathogenic variant in the *PTPRQ* gene, associated with non-syndromic sensorineural hearing loss.

## Treatment

Conventional treatment with oral phosphate supplements and calcitriol was initiated and titrated to maintain serum phosphate levels below the lower limit of normal, while improving alkaline phosphatase and symptoms. Rising PTH levels (up to 85 and 88 pg/mL in Twin 1 and Twin 2, respectively; reference <68.3) likely reflected phosphate-induced secondary hyperparathyroidism, which limited further escalation of oral phosphate. At present, both patients are receiving calcitriol (0.50 μg daily) and 1,000 mg of elemental phosphorus, divided into four doses throughout the day.

## Outcome and follow-up

After 1 year of treatment, both twins reported a marked improvement in symptoms and mobility. The previously wheelchair-dependent twin is now able to ambulate with a single crutch, and his brother regained independent walking. Both reported reduced bone pain, particularly in the lower limbs, improved mobility, and a marked enhancement in the quality of life.

## Discussion

Growth retardation, skeletal deformities, and chronic bone pain in children and young adults should prompt consideration of underlying metabolic bone disorders. Although nutritional rickets is the most recognized cause, the differential diagnosis is broad and includes hereditary forms of hypophosphatemic rickets, such as XLH, autosomal dominant hypophosphatemic rickets (ADHR), autosomal recessive forms (ARHR1/2), and other phosphate-wasting syndromes, such as tumor-induced osteomalacia (3). Despite hallmark features, such as short stature, genu varum, and recurrent dental abscesses, both patients remained undiagnosed into adulthood, underscoring how fragmented care and diagnostic inertia can delay appropriate work-up for rare inherited conditions such as XLH. Their clinical trajectory illustrates common pitfalls in the evaluation of skeletal disorders, particularly when symptoms are misattributed to nutritional deficiencies or isolated orthopedic conditions. Misdiagnosis can lead to delayed treatment and progression of complications. The need for coordinated input from endocrinology, orthopedics, neurosurgery, nephrology, and dentistry is often unmet, resulting in fragmented care and missed opportunities for early intervention. As emphasized in the literature (3), increasing awareness among non-specialist clinicians and primary care providers is essential to reduce diagnostic delays.

XLH remains the most prevalent hereditary form of rickets, yet its rarity, variable expressivity, and multisystemic manifestations often hinder timely diagnosis. In the absence of a positive family history – as in the present case – the diagnosis may be overlooked for years, especially in healthcare systems lacking integrated metabolic bone care. While XLH is typically inherited in an X-linked dominant pattern, up to 20–30% of cases result from *de novo* mutations, making it essential to consider this diagnosis even in the absence of familial involvement (4).

Following diagnosis, conventional treatment with oral phosphate supplements and calcitriol was initiated and titrated to improve skeletal mineralization and alleviate symptoms. Phosphate dosing was limited by rising parathyroid hormone levels – reflecting the risk of secondary hyperparathyroidism – and serum phosphorus levels remained well below the lower limit of normal (1.4 mg/dL in both twins, reference range: 2.7–4.5 mg/dL). Nevertheless, the clinical response was notable. After 1 year of therapy, both twins reported substantial reductions in bone pain and improved mobility. The previously wheelchair-bound twin regained partial ambulation with a crutch, while his brother was able to walk unaided.

Conventional therapy remains the cornerstone of XLH management in many parts of the world and is particularly valuable when initiated early. Treatment during childhood has been shown to reduce bone deformities, improve growth velocity, prevent pseudofractures, and delay long-term complications, such as osteoarthritis or enthesopathy (5). In contrast, delayed diagnosis and treatment often result in irreversible skeletal changes, chronic pain, functional impairment, and psychosocial consequences. Despite this, our patients demonstrated that even late initiation of conventional therapy can yield meaningful symptomatic improvement.

However, long-term phosphate and calcitriol therapy poses several challenges, including gastrointestinal intolerance, difficulties with adherence due to multiple daily doses, risk of secondary hyperparathyroidism, and the potential for nephrocalcinosis (5). These limitations have prompted the development of newer targeted therapies.

Burosumab, a fully human monoclonal antibody that inhibits excess FGF23, has recently emerged as a major advance in the treatment of XLH. By directly addressing the pathophysiological mechanism of the disease, burosumab normalizes phosphate levels without inducing secondary hormonal imbalances. Clinical trials have shown improvements in phosphate homeostasis, bone mineralization, fracture healing, physical performance, and quality of life in both pediatric and adult populations (6). It is now considered a first-line therapy in many guidelines. However, access remains limited in some countries and healthcare systems, particularly for adult patients.

In our case, burosumab was not initiated due to restricted access at the time of diagnosis. Nonetheless, the positive response to conventional therapy highlights its ongoing relevance, especially in resource-limited contexts and in adult patients with long-standing, untreated disease.

Genetic testing confirmed a novel hemizygous deletion affecting exon 22 of the *PHEX *gene: [c.(2,147 + 1_2,148−1)_(*408_?)del p?]. This variant affects the canonical splice donor site and is predicted to disrupt the normal mRNA processing. The pathogenicity of similar deletions in this region is well established, supporting its causal role in the clinical phenotype observed.

The remarkably similar clinical, biochemical and radiographic profiles – along with a comparable therapeutic response – observed in these genetically identical twins provide a unique perspective on the ongoing debate regarding genotype–phenotype correlations in XLH. While several studies have reported significant intra-familial and intra-genotypic variability, even among individuals carrying the same *PHEX* mutation (7), many have also found an overall lack of consistent genotype–phenotype correlation (8). Conversely, other studies suggest that more deleterious mutations, such as truncating variants, may be linked to more severe biochemical and clinical phenotypes, including lower renal tubular reabsorption of phosphate (TRP) and reduced 1,25(OH)_2_D levels (9).

In our case, the strong phenotypic concordance may indicate a significant pathogenic effect of the mutation. However, given the twins’ identical genetic background and shared environment, we cannot exclude the role of additional modulating factors. Notably, the presence of an Arnold–Chiari type I malformation in only one twin raises the possibility of additional epigenetic, developmental, or stochastic modifiers. Owen *et al.* described female monozygotic twins with discordant phenotypes – only one of whom was affected – but no pathogenic *PHEX* mutation was identified, complicating the interpretation (10).

This case report describes the first known instance of phenotypic concordance in monozygotic twins with XLH due to a novel *PHEX* mutation, offering a unique perspective on disease expression in a genetically and environmentally matched context. It highlights the diagnostic challenges posed by sporadic presentations and emphasizes the importance of a high index of suspicion in patients with unexplained growth failure, skeletal deformities, or recurrent dental problems.

Identification of a previously unreported *PHEX* variant further expands the known mutational spectrum of XLH and contributes to the ongoing efforts to understand genotype–phenotype variability in this complex disorder.

## Declaration of interest

The authors declare that there is no conflict of interest that could be perceived as prejudicing the impartiality of the work reported.

## Funding

This work did not receive any specific grant from any funding agency in the public, commercial or not-for-profit sector.

## Patient consent

Written informed consent for publication of their clinical details and/or clinical images was obtained from both patients.

## Author contribution statement

SR and TM conceptualized the report. SR, AV and GS conducted the clinical investigation. Data collection was performed by SR and AV. Formal analysis was carried out by SR, AV, GS and JQ. GS was responsible for the genetic analysis and interpretation. SR drafted the original manuscript. All authors (SR, TM, AV, GS, and JQ) contributed to the review and editing of the manuscript. Supervision was provided by SR and AV. All authors have read and approved the final version of the manuscript.
